# Hydrocele in the canal of nuck in a young female: a rare case report

**DOI:** 10.1093/jscr/rjac316

**Published:** 2022-07-05

**Authors:** Rajan Sood, Sourabh Trivedi, Paran Tanwar

**Affiliations:** Department of General Surgery, Maharishi Markandeshwar Medical College and Hospital, Solan, India; Department of General Surgery, Maharishi Markandeshwar Medical College and Hospital, Solan, India; Maharishi Markandeshwar Medical College and Hospital, Solan, India

## Abstract

Hydrocele of the canal of Nuck is a rare condition seen in younger females which is an extension of the peritoneum into the inguinal canal. Incomplete proximal obliteration and collection of serous fluid in the sac leads to the formation of a hydrocele of the canal of Nuck. A 28-year-old woman presented with swelling in her right groin for 5 months of 6cm × 5 cm. Ultrasound revealed a well-defined tubular cystic structure, measuring 5 cm × 3.5 cm × 5 cm with a volume of 50–60 ml. The cyst was dissected and the neck of the sac was extended up to the deep ring. Clear fluid was found on opening the sac. Clinically, it appears either as a painless or a moderately painful fluctuant inguinal mass. Clinical findings alone do not help in diagnosing the disease. Treatment includes surgical excision of the mass but without puncturing it as aspiration is inadequate and results in recurrence.

## INTRODUCTION

The round ligament of the uterus is fibro-muscular connective tissue, round band of rope in appearance. One side of the round ligament is attached to the superior and lateral aspect of the uterus. From the cornu of the uterus, the round ligament crosses the pelvis through the deep inguinal ring, which then traverses the inguinal canal and then enters the labia majora, where it terminates with its fibers blending into the mons pubis [1]. Folded peritoneum covers the round ligament, which traverses through the inguinal canal called the canal of Nuck, which is the counterpart of process vaginalis in males. It usually gets obliterated, but it leads to different clinical entities if it remains patent. One of which is encysted hydrocele of canal of Nuck, which is commonly present in pediatric age group, but our patient, presented in adulthood which is a rarity.

## CASE REPORT

A 28-year-old married woman presented with swelling in her right groin for 5 months. This swelling was initially of the size of a small lemon but gradually increased in size over 6 cm × 5 cm. There was no association with pain and other signs of subacute obstruction. The swelling was irreducible and fluctuant with no cough impulse and transillumination. The skin over the swelling was normal. Past history of being operated for bilateral ovarian cysts around 8 years ago was present by the lower midline incision; however, no histopathological report was available. Differential diagnosis of mesothelial cyst of the round ligament, cystic lymphangioma, epidermal inclusion cyst, cold abscess, varicosity of the round ligament, and endometriosis of round ligament was entertained.

**Figure 1:**
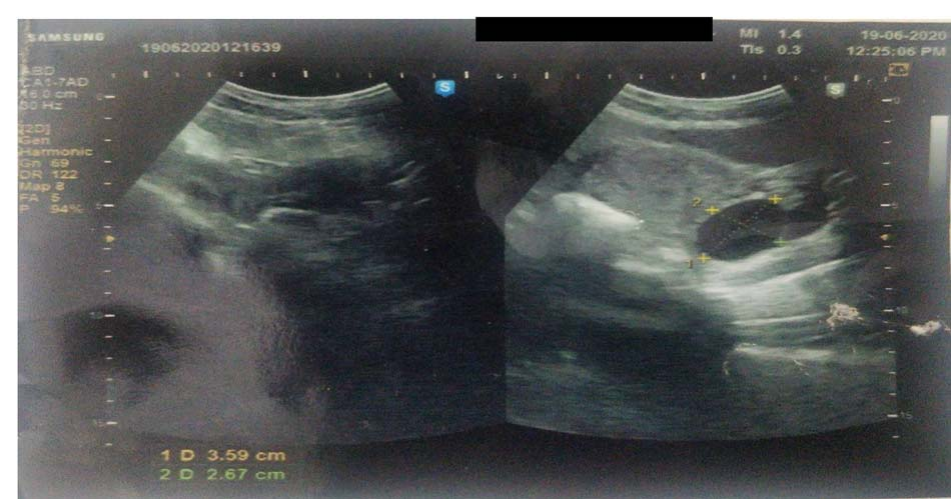
Ultrasound showing a well-defined tubular cystic structure with clear contents in the right inguinal canal up to labia majora measuring 5 cm × 3.5 cm × 5 cm with a volume of 50–60 ml.

Ultrasound revealed a well-defined tubular cystic structure with clear contents in the right inguinal canal up to labia majora measuring 5cm × 3.5 cm × 5 cm (Fig. 1) with a volume of 50–60 ml. Color doppler revealed avascular cystic structure. The size and contents of the cyst are not increasing on the Valsalva maneuver.

**Figure 2:**
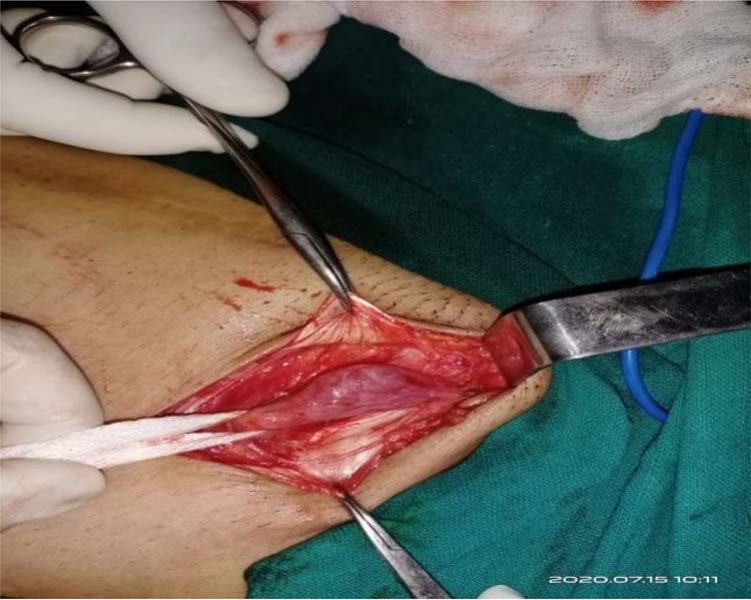
At surgery, through an inguinolabial incision, the cyst was dissected by blunt and sharp dissection from a round ligament in the inguinal canal; the neck of the sac was extending up to a deep ring.

**Figure 3:**
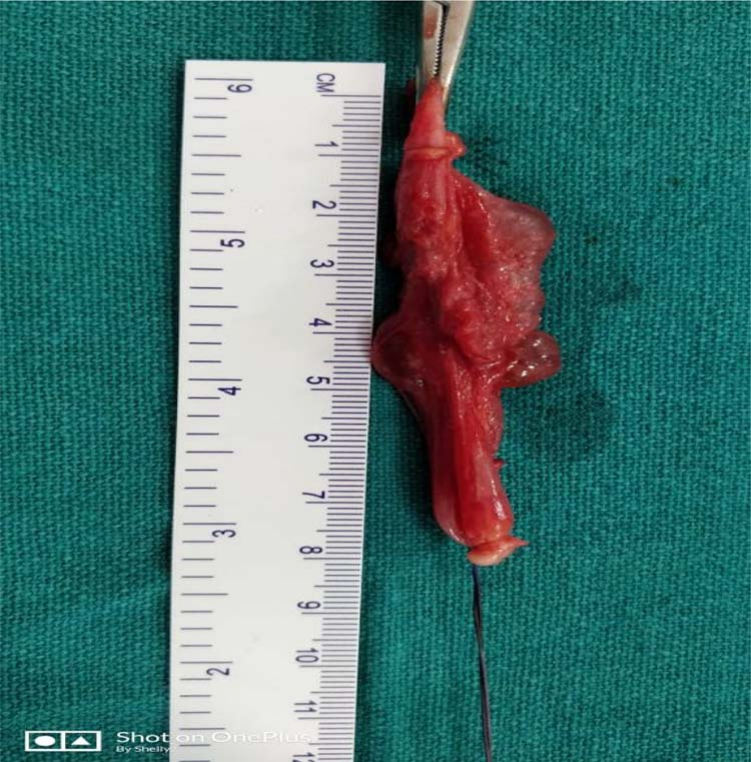
Showing sac measured about 5 cm.

At surgery, through an inguinolabial incision, the cyst was dissected by blunt and sharp dissection from the round ligament in the inguinal canal, and the neck of the sac was extended up to the deep ring (Fig. 2).The sac measured about 5 cm (Fig. 3). Clear fluid was found on opening the sac. Histopathology revealed flat mesothelial cells.

## DISCUSSION

The secretory lining of processus vaginalis produces peritoneal fluid, over secretion or under absorption, which may lead to the formation of cystic swelling. The etiological factors responsible for such cystic swelling are mostly idiopathic, and other causes are inflammation, trauma, impairment of lymphatic drainage and meconium hydrocoele [2]. Clinically, a hydrocoele of the canal of Nuck can appear either as a painless or a moderately painful fluctuant inguinal mass. These masses do not have any cough impulse, irreducible, and can be trans-illuminated if large enough. A traction test can be demonstrated in males for clinical confirmation but cannot be elicited in females. Clinical findings alone do not help in diagnosing the disease. When the peritoneal pocket remains completely patent, it forms a route for an indirect inguinal hernia. Partial proximal obliteration, which leaves the distal portion of the processus vaginalis open, creates the anatomic pre-requisite for a hydrocoele of the canal of Nuck [3–5]. Treatment includes surgical excision of the mass but without puncturing it as aspiration is inadequate and results in recurrence. When it is complicated by endometriosis, excision of both the mass and uterine round ligament is necessary [3, 5, 6].

## CONFLICT OF INTEREST STATEMENT

None declared.

## FUNDING

None.
